# Classifying and characterizing the development of adaptive behavior in a naturalistic longitudinal study of young children with autism

**DOI:** 10.1186/s11689-017-9222-9

**Published:** 2018-01-05

**Authors:** Cristan Farmer, Lauren Swineford, Susan E. Swedo, Audrey Thurm

**Affiliations:** 1 Pediatrics and Developmental Neuroscience Branch, National Institute of Mental Health, National Institutes of Health, Bethesda, MD 20892 USA; 2 Department of Speech and Hearing Sciences, Washington State University, Spokane, WA 99202 USA

**Keywords:** Autism spectrum disorders, Adaptive behavior, Longitudinal studies

## Abstract

**Background:**

Adaptive behavior, or the ability to function independently in ones’ environment, is a key phenotypic construct in autism spectrum disorder (ASD). Few studies of the development of adaptive behavior during preschool to school-age are available, though existing data demonstrate that the degree of ability and impairment associated with ASD, and how it manifests over time, is heterogeneous. Growth mixture models are a statistical technique that can help parse this heterogeneity in trajectories.

**Methods:**

Data from an accelerated longitudinal natural history study (*n* = 105 children with ASD) were subjected to growth mixture model analysis. Children were assessed up to four times between the ages of 3 to 7.99 years.

**Results:**

The best fitting model comprised two classes of trajectory on the Adaptive Behavior Composite score of the Vineland Adaptive Behavior Scale, Second Edition—a low and decreasing trajectory (73% of the sample) and a moderate and stable class (27%).

**Conclusions:**

These results partially replicate the classes observed in a previous study of a similarly characterized sample, suggesting that developmental trajectory may indeed serve as a phenotype. Further, the ability to predict which trajectory a child is likely to follow will be useful in planning for clinical trials.

**Electronic supplementary material:**

The online version of this article (10.1186/s11689-017-9222-9) contains supplementary material, which is available to authorized users.

## Background

Autism spectrum disorder (ASD) is typically life-long, with impairments stemming from core symptoms that present early in development [1, 2]. ASD is frequently associated with intellectual disability [3], a diagnosis which requires deficits in adaptive behavior. However, regardless of cognitive function, individuals with ASD display deficits in adaptive functioning, both in the domains most directly affected by the core symptoms of ASD (e.g., socialization and communication) [4] and also more generally [5]. As such, adaptive functioning deficits have long been used to quantify the impairments in functioning required for the ASD diagnosis [6, 7] and to track changes in functioning [8], including in the early years [9] (see [10] for a historical review in individuals with ASD and intellectual disability).

Adaptive function has been discussed as a promising outcome measure for a variety of neurodevelopmental and neuropsychiatric conditions [11–14] because it has clinical significance for both families and researchers [15]. Adaptive behavior has been used rarely as a primary outcome in treatment research (e.g., [16]), though it appears listed amongst secondary outcomes in a number of trials. These include double-blind, placebo-controlled drug studies [17, 18] as well as studies that include behavioral interventions [19]. Some of these studies used independent evaluators, blind to treatment group, to conduct the interviews with parents or caregivers [20, 21], while others use the parent rating form in an unblinded fashion (e.g., [22]). In fact, there has been enough interest that researchers are investigating the best statistical methods for detecting change in adaptive functioning for future trials [23].

Most of our knowledge about adaptive behavior in individuals with ASD comes from cross-sectional studies, which suggest ASD-specific profiles that vary with factors such as age and IQ [15]. However, recently published data are making increasingly apparent that some phenotypic characteristics may be less stable over time than previously assumed [24]. This is based on the slower-than-expected, but not negligible, growth in skills as children age. Thus, it may not be the snapshot-in-time that best describes an individual, but rather his or her change over time; in other words, developmental trajectories themselves could serve as phenotypes [25].

We know less about the development of adaptive behavior in ASD than we do about its snapshot-in-time presentation, especially across longer periods during early to middle childhood. This dearth exists because there have been very few longitudinal studies of ASD beginning in the preschool period, and even fewer that report on longitudinal measurement of adaptive functioning. Available data indicate that on average, adaptive behavior deficits seem to persist into adulthood (for a review see [8]), and adaptive behavior in ASD is heterogeneous and variable, even within an individual. While adaptive behavior impairment has a generally predictable relationship with cognitive ability in samples of individuals with intellectual disability, the relationship appears to be more complicated in individuals with ASD. Children with ASD who do not have intellectual disability may still have impaired adaptive function [5], while individuals with both ASD and intellectual disability may have relatively less impairment in adaptive function compared to their level of cognitive impairment [26].

Some longitudinal studies have attempted to parse samples based on this heterogeneity, though for the most part, their sample sizes were small, they used few assessment points or limited age ranges, and/or they were focused on very specific domains of adaptive behavior (see Table 1). One useful statistical method for the empirical description of heterogeneous data is growth mixture models (GMM), which provide a richer understanding of the data than do standard growth curve models [27]. The most basic form of these models, latent class growth curve analysis (LCGA, known by other names, such as a “semi-parametric and group-based approach” and by the name of the program often used to implement it, Proc Traj), has been used in a handful of investigations of *within-subject* adaptive behavior development in ASD. In one study, investigators analyzed three assessments (5, 8, and 15 years of age) from 152 individuals with ASD, finding evidence for two patterns of development in age equivalents of adaptive behavior domains: one with little growth across the time points, and the other with substantial but less-than-expected growth [28]. Another study of approximately 85 individuals, assessed between the ages of 2 and 19 years, revealed the same two patterns of development, this time in the daily living skills domain [29]. Using an overall standard score measure of adaptive behavior, findings from four assessments of an inception sample of 406 children with ASD suggested low/worsening, moderate/stable, and average/improving trajectories over the period of 3 to 6 years of age [30].

**Table 1:** Vineland trajectory study summaries

Report	ASD, *n*	Age (years) at baseline	Length of follow-up (occasions)	Cognitive ability level at baseline	Summary of findings
Szatmari et al. [30] (overlaps with Flanagan et al. [42])	421	3.32 ± 0.75	Four assessments: baseline, 6 and 12 months post-baseline, and age 6 years	Merrill-Palmer-Revised Developmental Index (full-scale IQ): 57.23 ± 26.20	Three classes of ABC trajectory: lower/worsening, moderate/stable, and higher/improving
Anderson et al. [26]	144	2.46 ± 0.39	Six assessments at approximate ages of 2, 3, 5, 9, 18, and 21 years (plus parent report at 10 and 13 years) (not all time points used in all publications)	Non-verbal IQ: 62.4 ± 17.36	Outcome was Vineland socialization age equivalent. Two classes were observed for both groups. Autism—low and flat, and moderate with age-appropriate growth. PDD—moderate with faster than expected growth, and low with moderate growth
Bal et al. [32]	Autism: 93 PDD: 51	Autism: 2.43 ± 0.42 PDD: 2.43 ± 0.47		Mullen Scales of Early Learning Non-verbal mental age: 1.62 ± 0.56 years	Two classes of daily living skills age equivalents trajectory: high and low. While both gained skills over time, the low group gained at a slower rate.
Baghdadli et al. [28]	152	4.9 ± 1.3	Three assessments at approximately 5, 8, and 15 years	Did not use standard assessments. “cognition related to object (months)”: 22.4 ± 11.9; “Cognition related to person (months)”: 19.2 ± 10.8	Across the subdomains of adaptive behavior, two patterns of development in age equivalents were observed: one with little growth across the time points and the other with substantial but less-than-expected growth.
Current study	105	4.24 ± 1.30	Follow-up at 6-month intervals prior to the third birthday; annual follow-ups until 3 years of study participation	Full-scale developmental quotient: 49.88 ± 16.83	Two classes of ABC trajectory: low/decreasing, moderate/stable

*PDD* pervasive developmental disorder, not otherwise specified, *ABC* Adaptive Behavior Composite

### Current study

The goal of this longitudinal study (NCT00298246) was to identify subtypes of ASD based on medical and behavioral phenotypes. Adaptive behavior was a key construct which we expected to differentiate the participants, but this specific analysis was not proposed a priori. Rather, we set out to replicate and extend previous research on the heterogeneity of adaptive behavior in individuals with ASD, using more advanced statistical models and a study population unique in its age at assessment, density of assessments, and length of follow-up. In the current analysis, we use GMM to explain the heterogeneity in development of adaptive behavior in children with ASD. We hypothesized that this mixture model would better fit the data than a standard latent curve model, suggesting that variability in trajectories is better explained by two or more subpopulations, rather than one.

## Methods

### Participants and procedures

Informed consent for participation was obtained from the parents or legal guardians of participants, who were enrolled in a longitudinal natural history study of autism approved by an NIH Institutional Review Board (06-M-0102). Participants were recruited from the community based on diagnosed or suspected ASD. Recruitment sources included medical, educational, and other service providers, as well as general announcements. The study period was between 2006 and 2014. The primary inclusionary criterion was a DSM-IV-TR [1] diagnosis of autistic disorder, based on the gold standard diagnostic battery described below. Exclusionary criteria for this study were a primary language other than English, cerebral palsy, or unmanageable behavior problems that prevented participation in standardized testing procedures. A total of 106 participants with ASD were enrolled. Smaller groups with non-ASD developmental delay and typical development were enrolled but are not reported here.

The design of the study was accelerated longitudinal; at enrollment, participants were between the ages of 18 months and 7 years, exclusive (mean ± SD = 4.05 ± 1.28 years). Visits prior to the third birthday were spaced at 6-month intervals, and later visits were annual until the child completed at least 3 years of participation or until the child’s fifth birthday. For this analysis, data were restructured into a “wide” format (i.e., 1-year bands starting at 24 months). If an individual had more than one visit per age band, the earlier visit was retained.

### Measures

Participants were evaluated by expert doctoral-level clinicians who met research reliability standards on the Autism Diagnostic Interview-Revised (ADI-R; [31]) and the Autism Diagnostic Observation Schedule (ADOS; [32]). The diagnosis of autistic disorder was made using the information from these instruments, as well as the DSM-IV-TR.

At each visit, participants were administered a test to assess cognitive ability, either the Mullen Scales of Early Learning [33] or the Differential Abilities Scales, Second Edition [34]. To facilitate comparison between the tests and to account for the inability of participants to achieve standard scores, we use developmental quotients (DQ; the ratio of mental age to chronological age) in place of conventional IQ.

Parents responded to several interviews and questionnaires, including the Child Behavior Checklist (CBCL; [35]) and the interview version of the Vineland Adaptive Behavior Scales, Second Edition (VABS; [36]). The VABS is a semi-structured interview that assesses adaptive behavior in several domains, summarized by the Adaptive Behavior Composite (ABC) standard score. ABC standard scores may range from 20 to 160, with a population mean of 100 and a standard deviation of 15. To facilitate comparison with existing studies, we used the ABC as our outcome measure.

This battery was repeated at all visits, excepting the ADI-R, which was conducted only at the first and last visits. Because the age band at study entry differed across participants, we could not evaluate baseline predictors of class membership. Instead, we plot observed contemporaneous data across several domains of interest (non-verbal and verbal DQ, ADOS Calibrated Severity Score (CSS), and CBCL Externalizing and Internalizing scores) by most likely class assignment.

### Statistical analysis

We used GMM to evaluate the developmental trajectory of adaptive behavior in children with ASD and to characterize the heterogeneity in these trajectories. While this analytic approach has been commonly employed in other areas of the developmental literature, there have been limited applications in the developmental disability literature. Further, the method we used is more extensive and complete than previously published in the ASD literature (e.g., [28–30]). For this reason, we present a brief overview (for more in-depth and technical introductions, see [37, 38]) and we provide the necessary Mplus syntax in Additional file 1.

GMM is an extension of conventional latent growth modeling, a class of statistical procedures used in longitudinal investigations to characterize both intra- and inter-individual variability in change. GMM may be particularly helpful in testing the assumption that the parameters from a standard growth model adequately describe data from two subpopulations (e.g., in physical growth curves, sex would be a known marker of subpopulation), especially when the explanation for heterogeneity is unknown. GMM treats this unknown as a latent variable problem, explained by an unobserved class variable. GMM provides information about whether the observed data are best explained by a single distribution of trajectory parameters (i.e., a latent class variable with only one class) or by a mixture of component distributions (i.e., a latent variable with two or more classes) [38].

Because the goal of GMM is to determine whether the data are best explained by one or more distributions, the first step is to establish the latent growth model (i.e., the best fitting model, assuming that there are no subpopulations reflected in the data). Subsequent GMM models will be compared to this “baseline” model to determine whether assuming a mixture of distributions, rather than a single distribution, improves fit.

GMMs of increasing complexity are then fit to the data. These models are distinguished by which parameters (i.e., mean, variance, and covariance of the intercept, slope, and/or quadratic terms) are allowed to vary, both within and between the classes. The simplest GMM is the latent class growth analysis (LCGA), which estimates only the mean values of the intercept, slope, and quadratic terms. These parameters are allowed to vary *between* classes, but not *within* (i.e., the variances, and therefore covariances, are constrained to zero). This means that all members of class 1 are constrained to have the same intercept, for example, but that intercept differs from those of the members of class 2. While it is possible that this preliminary model is appropriate, whether the variances and covariances should be constrained to zero is an empirical question. Thus, the remaining procedures entail the evaluation of at least four more models in the following sequence: (a) relax the within-class constraint on the variance of the intercept and slope factors (GMM1), (b) relax the within-class constraint on the covariance of the intercept and slope factors (GMM2), (c) relax the between-class constraint on the variance of the intercept and slope factors (GMM3), and (d) relax the between-class constraint on the covariance of the intercept and slope factors (GMM4). Each model specification is then evaluated for one, two, three, four or more classes, or until the model is no longer able to converge.

The best model is selected in an iterative process. First, the relative fit indices of all models are compared. In the current analyses, we used the following fit indices: the loglikelihood, the Bayesian information criterion, the adjusted Bayesian information criterion, Aikake’s information criterion, and the consistent Aikake’s information criterion. Bayes’ factor and the approximate weight of evidence criterion were used to assist in the interpretation of information criteria. Finally, the Vuong-Lo-Mendell-Rubin likelihood ratio test (and an adjusted value) and the parametric bootstrap likelihood ratio test were used to assess the degree of improvement in model fit with additional classes. Each of these fit indices is described in Additional file 1.

Next, a handful of candidate models with the best profile of relative fit indices are further evaluated based on their classification quality and the degree of distinction between classes. In this study, we calculated entropy, the average posterior probability, the odds of correct classification, and the modal class assignment proportion for each class. The homogeneity and separation were calculated for each parameter that was allowed to vary and therefore characterize each class. The models were also evaluated for robustness to slight changes in the model specification; for example, does class assignment in the two-class solutions change significantly between the GMM1 and GMM2 specifications?

All GMM analyses were completed in Mplus version 7.4; other analyses and data management were performed in SAS/STAT version 9.3. We note that the maximum likelihood estimation with robust standard errors accommodates the missing data imposed by the age-cohort structure of the data. Because the results and fit indices for all 21 models are voluminous, they are reported in Additional file 1.

## Results

Sparseness of data at age bands 2, 8, and 9 (see Additional file 1: Figure S1) was likely to cause convergence problems, so only age bands 3 through 7 (representing visits between the ages of 3 to 7.99 years) were used in this analysis. All 106 participants had at least one visit within these age bands, but we made the a priori choice to exclude from analysis one participant with data in age bands 3 and 4, who was an outlier with abnormally high ABC scores compared to other participants in the sample (see Additional file 1: Figure S2). Baseline demographic information, obtained at the first visit included in this analysis (not necessarily the participant’s first study visit), for the remaining 105 participants is shown in Table 2. The number of study visits per participant ranged from one to five (median = 4) (see Additional file 1: Figure S1 for data coverage). Seven participants had only one visit.

**Table 2:** Participant demographics at baseline (*n* = 105)

	*n* (%)	Mean	Standard deviation
Male	91 (88)		
Age (years)	105 (100)	4.24	1.30
Maternal education			
High school	10 (10)		
Some college/college degree	63 (60)		
Graduate degree	28 (27)		
Not reported	4 (4)		
Full-scale developmental quotient	103 (98)	49.88	16.83
Non-verbal developmental quotient	103 (98)	58.39	16.87
Verbal developmental quotient	103 (98)	41.01	18.49
ADOS Calibrated Severity Score	103 (98)	7.66	1.40
Vineland Adaptive Behavior Composite	105 (100)	65.55	8.88
Full-scale DQ and Vineland ABC < 70	67 (64)		

The age cohorts 2, 8, and 9 were excluded from analysis and are therefore not reflected in this table. Thus, baseline in these analyses was not the first visit for all participants

Consistent with visual inspection, the best-fitting latent growth model was of quadratic form, where the variance in both the intercept and slope was estimated but was constrained to zero for the quadratic term. We used this baseline model for the remaining GMM analyses.

The full complement of fit indices for the GMM specifications is shown in Additional file 1: Table S1. Based on these results, the candidate models were the LCGA two-class, LCGA three-class, and the two-class solutions in the GMM1, GMM2, and GMM3 parameterizations. First, we reviewed the parameter estimates from each candidate model (Additional file 1: Table S2). The GMM parameterizations differ from LCGA in that they allow variation within the group on the intercept and slope factors. The within-class variance of the intercept, but not the slope, was large and significantly different from zero, suggesting that the GMM parameterizations might be more reflective of the data than the LCGA. However, the covariance between the intercept and slope, which was allowed to be non-zero in the GMM2 parameterization, was not significant. Further, when the between-class variance was allowed in intercept and slope (GMM3), these parameters were non-significant. Considering that the mean estimates for the intercept and slope were similar for each class across the GMM parameterizations, the GMM1 appeared to be the best representation of the data.

Next, classification quality, as well as the homogeneity and separation of the resulting classes, was evaluated for the LCGA and GMM1 parameterizations (Additional file 1: Table S3). All models had acceptable classification quality, homogeneity, and separation. Thus, the selection of the GMM1 two-class solution, rather than the LCGA solution, was driven by the large and significant within-class variance of the intercept.

**Fig. 1:**
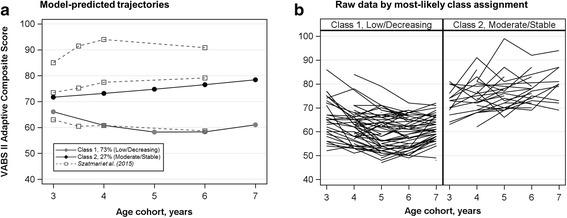
Final GMM solution. **a** The estimated proportion of class membership was 73% for class 1 and 27% for class 2. The slope and quadratic terms were significant for class 1, but not for class 2 (see Additional file 1: Table S2 for parameter estimates). The trajectories observed by Szatmari et al. [30], by modal class assignment, are superimposed with dotted lines. **b** Observed ABC scores by most likely class assignment

In the final model (Fig. 1), the low/decreasing class (class 1) was characterized by an ABC score of approximately 66 at age 3 years and a significant quadratic trajectory. The moderate/stable class (class 2) was characterized by a slightly higher age 3 score (about 72), with no change over the study period (i.e., slope and quadratic terms were non-significant) (see Additional file 1: Table S2). The model-estimated proportion of the sample in each class was 73 and 27%, respectively. To facilitate the comparison to published data, the observed means from the Szatmari et al. study were superimposed on the current study estimated class trajectories in Fig. 1.

**Fig. 2:**
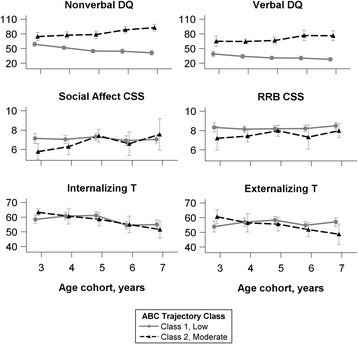
Phenotypic data over time, by most likely class assignment. Mean (95% confidence interval) scores on cognitive tests, ADOS CSS, and CBCL are shown for each class. Most-likely assignment was class 1 for 76% of the sample and class 2 for 24%. Sample size for each age cohort varies (see Additional file 1: Table S4)

The classes are descriptively characterized using other phenotypic data in Fig. 2. Modal class assignment was used to calculate the mean cognitive, ADOS CSS, and CBCL scores. Stronger non-verbal DQ relative to verbal DQ was characteristic of both classes, and in both classes, more change was observed over time in non-verbal DQ than in verbal DQ. However, for the moderate/stable class, average DQ scores increased over time, while average DQ scores decreased over time for the low/decreasing class. No characteristic patterns were observed in ADOS CSS scores, which remained relatively stable over time, nor were the classes distinguished by CBCL Internalizing or Externalizing scores, though a general trend of decreasing over time was observed in both classes for the former.

## Discussion

We used longitudinal data from children with ASD aged 3 to 7.99 years to explore heterogeneity in the development of adaptive behavior. About three quarters of the sample were assigned to a low/decreasing trajectory. The remainder of the sample were best classified in a trajectory exemplified by stable scores around 70 (moderate/stable). Thus, even within a sample likely to exhibit a high rate of intellectual disability later in childhood, we observed variability in the progression of adaptive function over time. Still, these data confirm previous findings that on average, young children with ASD are likely to exhibit significantly impaired adaptive function (based on age-referenced standard scores) during the preschool to school-age period, with only a minority exhibiting an improving trajectory [23, 30].

These two trajectories are reminiscent of patterns observed in studies of the development of cognitive ability; on average, children with more moderate scores tend to improve somewhat over time, while children with lower scores appear to fall further behind. The latter is to be expected with the use of standard scores; anything less than on-pace gains in skills will result in decreasing standard scores over time. Decreasing standard scores over time are certainly not unique to ASD; similar trajectories have recently been described in various genetic disorders associated with intellectual disability, including fragile X syndrome, Williams syndrome, and tuberous sclerosis. This suggests that the presence of intellectual disability, rather than ASD specifically, may be the predominate explanatory factor for these declines. It is also possible that this pattern is reflective of psychometric properties of the VABS; with relatively fewer items at the lower extremes, stability in standard scores is difficult to achieve.

Adaptive behavior development does not exist in a vacuum, so it is essential to characterize subpopulations in terms of other phenotypic characteristics. Other studies have documented consistently that lower cognitive and language ability predicts less optimal trajectories of adaptive behavior [28–30]. This finding is echoed by data from cross-sectional or pre-post analytic designs (e.g., [9, 39, 40]). Given the strong correlations between measures of IQ and measures of adaptive behavior, it is unsurprising that changes in non-verbal and verbal DQ paralleled the adaptive behavior trajectory in our study. While IQ is not the only determinant of adaptive behavior, and studies have shown discrepancies between adaptive behavior scores and IQ scores may depend on IQ range [26, 41], it is well-established that cognitive impairment negatively affects the ability to carry out functions of daily life, above and beyond the effects of symptoms of ASD. However, while there was little difference in adaptive behavior and cognitive scores in the moderate/stable class, the average cognitive score in the low/decreasing class was much lower than the adaptive behavior score. This profile of relatively stronger adaptive behavior, compared to IQ, has been observed in other samples of individuals with low IQ [15, 42]. However, when IQ is not in the range of intellectual disability, adaptive behavior is often found to be lower than IQ in ASD [5], leading to the suggestion that the pattern is driven by deficits in social abilities [43]. The high proportion of children with low IQ and/or language impairment in our sample necessitated the use of ratio IQ scores, and this may have influenced our results by overemphasizing the effect of age (ratio IQs are divided by an ever-increasing denominator of chronological age). This may artificially delate IQ, resulting in lower ratio IQs than adaptive behavior scores in those with the lowest IQs.

The finding that the development of VABS composite scores over the preschool and early school-age years in our sample was best described by low/decreasing and moderate/stable classes was remarkably consistent with findings reported by Szatmari et al. and may be considered a partial replication, enhancing our confidence that these subpopulations exist. The wider age range in our study extends the Szatmari et al. findings, although a longer follow-up with denser sampling is necessary to confirm the short-term stabilizing trend observed after the age of 6 years in these data. In our sample, we did not find evidence for the “high functioning and improving” class described by Szatmari et al., which may reflect several factors, including ascertainment (they included participants referred to the longitudinal study directly from community referral centers, whereas our participants were mostly self-referral from the community) and diagnosis (they included DSM-IV-TR pervasive developmental disorder, not otherwise specified, while we required autistic disorder). Given that our sample had few participants with cognitive scores in the average range, we surmise that we simply did not sample the population represented by the “high functioning and improving” class in Szatmari et al. [30]. However, we do note that while the average IQ score in our sample was lower than that of Szatmari et al., mean IQs in both studies indicate similar levels of intellectual disability. Our slightly lower IQ likely reflects the more severely impaired cognitive profile of children already diagnosed with DSM-IV-TR autistic disorder (as opposed to an inception sample including pervasive developmental disorder, not otherwise specified). Thus, because our sample did not include many children with average IQs, these results are only generalizable to the subset of the ASD population with low IQ scores.

These data help to address the dearth of longitudinal natural history data on adaptive behavior in children with ASD and generally low cognitive ability. The most serious weaknesses of this study are its relatively small size, especially at the more extreme ages, and our inability to evaluate early predictors of class membership due to varying ages of study entry. Although we used the semi-structured survey interview form of the VABS, the necessary reliance on parent report may have biased results. However, a significant strength of this study is the analytic approach. While not novel in the broader child development literature, GMM has been implemented in few ASD studies, and when they have been used, researchers were likely to stop at the LCGA specification (e.g., [28–30]), limiting insights into data patterns that may not be obvious with traditional growth models.

Finally, we underscore the importance of these findings in relation to the ongoing search for appropriate outcome measures in intervention trials. Due to an emphasis on function as the most important outcome, adaptive behavior may become a more common primary and secondary target in clinical trials [44, 45]. We confirmed the presence of at least two subpopulations of adaptive behavior trajectories within ASD—children who are moderately impaired, but exhibit stable adaptive behavior standard scores over the early childhood and school-age years, and those who have more impaired scores that worsen over time. The reliable identification of the latter class would be advantageous for any clinical trial that uses adaptive behavior as an outcome. Specifically, researchers are investigating novel statistical methods to identify a “minimal clinically important difference” [23] in order to ease reliance on classical null hypothesis testing wherein significance is defined only by the difference from zero. Importantly, in some cases, this may actually be manifested as stability, or just minimal improvements in adaptive behavior standard scores (but growth in raw scores). The degree of meaningfulness may depend upon ability level [23] and may be further adjusted based on membership in an adaptive behavior trajectory subpopulation like those described herein. Szatmari et al. found that language and cognitive scores predicted class membership; it will be important to also explore whether the initial level of adaptive behavior had similarly predictive power. Future research, focused on the identification of predictors of membership, will help to translate descriptive findings into clinically and empirically useful information.

## Conclusions

In this analysis, we reported data from one of the few longitudinal studies of ASD to include the transition from preschool to school-age, replicating with sophisticated statistical modeling the general findings of previous studies examining the development of adaptive behavior. These findings illustrate that early delays in adaptive behavior are stable or worsen from the preschool to school-age periods for the majority of children enrolled in these research cohorts, characterized by growth in adaptive behavior skills that lags behind the change in chronological age. For some children with lower adaptive abilities, this slower-than-expected growth results in a decline in composite standard scores during childhood. These findings provide critical context for the interpretation of changes in adaptive behavior scores in clinical trials.

## Additional file

Additional file 1: **Figure S1.** Data coverage. **Figure S2.** Raw ABC scores. **Table S1.** Model fit indices. **Table S2.** Model information. **Table S3.** Classification quality. **Table S4.** Correlates of class membership. **Table S5.** Mplus syntax for growth mixture models. (PDF 778 kb)

## Abbreviations

ABC: Adaptive Behavior Composite
ADI-R: Autism Diagnostic Interview-Revised
ADOS: Autism Diagnostic Observation Scale
ASD: Autism spectrum disorder
CBCL: Child Behavior Checklist
DQ: Developmental quotient
GMM: Growth mixture model
IQ: Intelligence quotient
LCGA: Latent class growth analysis
PDD: Pervasive developmental disorder not otherwise specified
VABS: Vineland Adaptive Behavior Scales
