# Supplementary material for: Classifying and characterizing the development of adaptive behavior in a naturalistic longitudinal study of young children with autism
Source: J Neurodev Disord. 2018 Jan 5;10:1. doi: 10.1186/s11689-017-9222-9 (PMC5795287; doi:10.1186/s11689-017-9222-9)
Supplement: Additional file 1: — Figure S1. Data coverage. Figure S2. Raw ABC scores. Table S1. Model fit indices. Table S2. Model information. Table S3. Classification quality. Table S4. Correlates of class membership. Table S5. Mplus syntax for growth mixture models. (PDF 778 kb) [file 11689_2017_9222_MOESM1_ESM.pdf]

List of Supplementary Figures and Tables

Figure 1	Data Coverate
Figure 2	Raw ABC scores
Table 1	Model Fit Indices
Table 2	Model Information
Table 3	Classification Quality
Table 4	Correlates of class membership
Table 5	Fit Index interpretation
Mplus syntax	

Figure 1. Data coverage. Each line represents an individual (*n* =106) and each dot represents a visit. Data were restructured into "wide" format (COHORT=age band); visits that occurred between 2.0 and 2.99 years of age were categorized into age band 2, those between 3.0 and 3.99 into age band 3, and so on. When a participant had more than one visit per age band, only the earlier visit was retained. Due to non-convergence in the models, age bands 2, 8, and 9 were excluded from analysis.

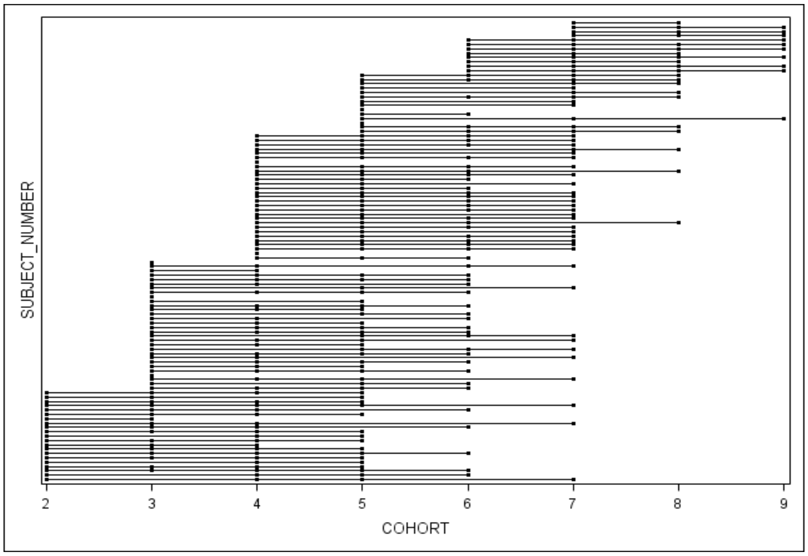

Cohort (Age Band)	Subjects with data
**2**	20
**3**	46
**4**	68
**5**	79
**6**	58
**7**	55
**8**	21
**9**	10

Figure 2. ABC Scores in Full Sample (N=106). Each line represents an individual and each dot represents a visit. Data were restructured into “wide” format; visits that occurred between 2.0 and 2.99 years of age were categorized into age band 2, those between 3.0 and 3.99 into age band 3, and so on. When a participant had more than one visit per age band, only the earlier visit was retained. Subject with abnormally high ABC values was excluded from analysis.

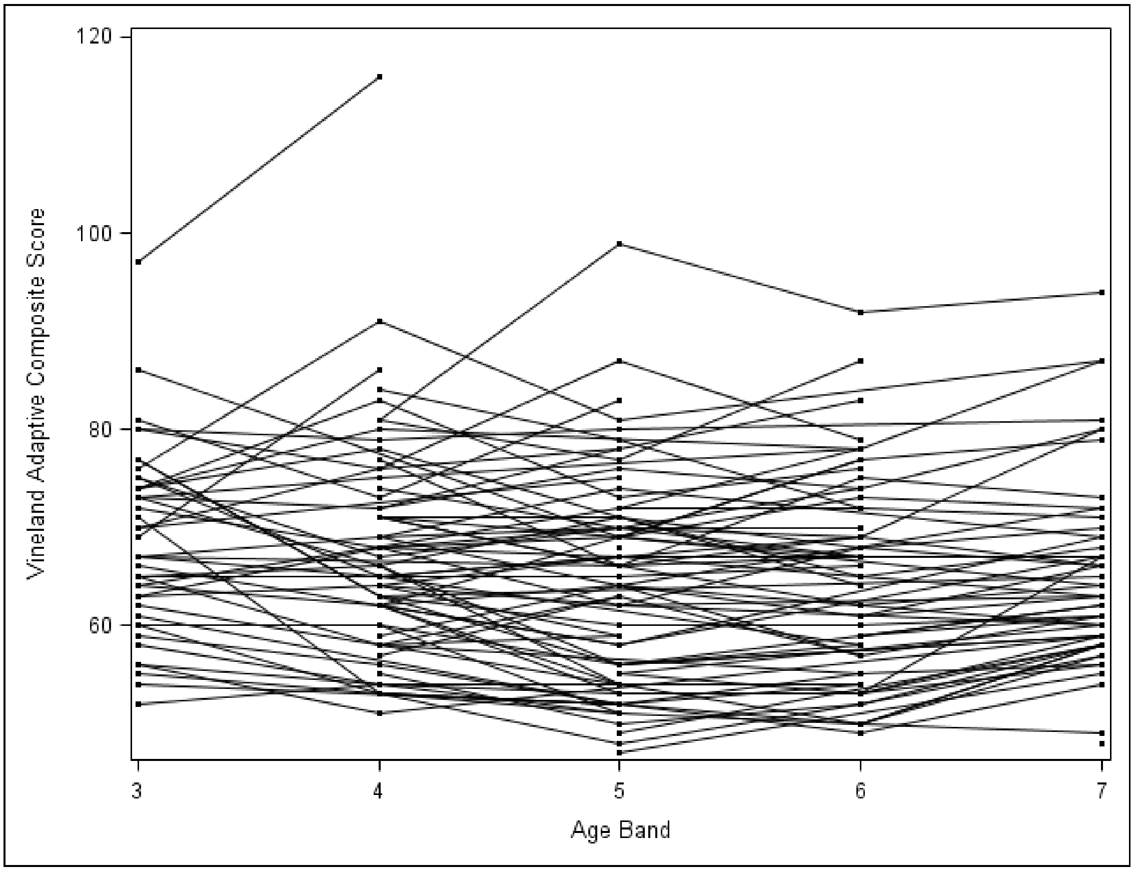

Table S1. Model Fit Indices

ABC Standard Score, Quadratic	CLASSES	STARTS	FINAL STARTING VALUE SETS CONVERGING, N(%)	LL REPLICATION, N(%)	SMALLEST CLASS, N(%)	CONDITION NUMBER	N class=1	N class=2	N class=3	N class=4	N class=5	# Parameters	Loglikelihood	AIC	BIC	ABIC
LCGA	1	500/100	500	100 (100%)	n/a	3.96E-05	105	n/a	n/a	n/a	n/a	8	-1123.23	2262	2283	2285
	2	500/100	500	100 (100%)	40.7 (38.8%)	2.31E-05	39	66	n/a	n/a	n/a	12	-1045	2115	2147	2109
	3	500/100	500	100 (100%)	15.98 (15.2%)	2.77E-05	38	52	15	n/a	n/a	16	-1008.98	2049	2092	2041
	4	500/100	500	59 (59%)	11.43 (10.9%)	2.74E-05	34	32	29	10	n/a	20	-990	2020	2073	2010
GMM1*	1	500/100	500	100 (100%)	n/a	1.00E-03	105	n/a	n/a	n/a	n/a	10	-1001	2020	2044	2015
	2	500/100	500	44 (44%)	28.6 (27.3%)	1.85E-04	80	25	n/a	n/a	n/a	13	-979	1984	2018	1977
	3	500/100	500	77 (77%)	24.8 (23.7%)	1.71E-04	55	25	25	n/a	n/a	17	-973	1981	2026	1972
	4	500/100	500	29 (29%)	1.90 (1.8%)	8.03E-08	23	26	54	2	n/a	21	-968	1978	2034	1967
GMM2	1	500/100	500	100 (100%)	n/a	2.19E-04	105	n/a	n/a	n/a	n/a	11	-995	2012	2041	2006
	2	500/100	500	29 (29%)	27.2 (25.9%)	1.14E-04	25	80	n/a	n/a	n/a	15	-977	1985	2024	1977
	3	500/100	500	17 (17%)	16.0 (15.2%)	7.52E-05	68	10	27	n/a	n/a	19	-972	1983	2033	1973
	4	500/100	500	20 (20%)	1.96 (1.9%)	4.85E-08	22	54	2	27	n/a	23	-967	1981	2042	1969
GMM3*	1	500/100	500	100 (100%)	n/a	1.69E-04	105	n/a	n/a	n/a	n/a	9	-1001	2020	2044	2015
	2	500/100	461	5 (5%)	28.56 (27%)	1.69E-04	24	81	n/a	n/a	n/a	14	-979	1986	2023	1979
	3	500/100	381	2 (2%)	28.51 (27.1%)	2.35E-06	25	48	32	n/a	n/a	19	-969	1976	2027	1967
	4	500/100	274	1 (1%)	8.74 (8.3%)	1.12E-05	5	51	26	23	n/a	24	-963	1975	2039	1963
GMM4	1	500/100	500	100 (100%)	n/a	2.19E-04	105	n/a	n/a	n/a	n/a	11	-995	2012	2041	2006
	2	500/100	364	1 (1%)	43.0 (40.9%)	7.88E-05	65	40	n/a	n/a	n/a	18	-973	1982	2029	1972
	3															
	4															

Table S1. Model Fit Indices

ABC Standard Score, Quadratic	Vuong-Lo-Mendell-Rubin LRT p-value	Lo-Mendell-Rubin adjusted LRT p-value	Parametric Bootstrap LRT p-value	SIC	Bayes' Factor	AWE	CAIC	Notes
LCGA	n/a	n/a	n/a	-1141.5	n/a	2344.92	2291.69	
	0.086	0.095	0	-1073.5	3.40428E+29	2237.70	2157.85	
	0.16	0.17	1	-1046	8.77199E+11	2214.89	2108.42	
	0.3	0.32	1	-1036.5	13359.72683	2226.16	2093.08	
GMM1*	n/a	n/a	n/a	-1022	n/a	2125.08	2058.54	* (Only Intercept is freed, because freed slope caused Psi matrix to not be positive definite).
	0.005	0.006	0	-1009	442413.392	2118.00	2031.50	
	0.46	0.48	1	-1013	0.018315639	2155.23	2042.12	
	0.05	0.05	1	-1017	0.018315639	2194.47	2054.73	
GMM2	n/a	n/a	n/a	-1020.5	n/a	2125.39	2052.19	
	0.02	0.03	0	-1012	4914.76884	2138.62	2038.81	
	0.33	0.35	0.67	-1016.5	0.011108997	2177.85	2051.43	
	0.2	0.21	0.67	-1021	0.011108997	2217.08	2064.04	
GMM3*	n/a	n/a	n/a	-1022	n/a	2112.77	2052.89	* (Only Intercept is freed, because freed slope caused Psi matrix to not be positive definite).
	0.008	0.01	0	-1011.5	36315.50267	2130.31	2037.16	
	0.12	0.14	0.67	-1013.5	0.135335283	2171.85	2045.43	
	0.38	0.4	0.6	-1019.5	0.002478752	2221.39	2061.70	
GMM4	n/a	n/a	n/a	-1020.5	n/a	2125.39	2052.19	
	0.23	0.24	0	-1014.5	403.4287935	2167.54	2047.77	Psi matrix is not positive definite. The correlation between I and S is >1 for both classes. The variance of S is not significantly different from zero in either class, so model not pursued.

Table S1. Model Fit Indices

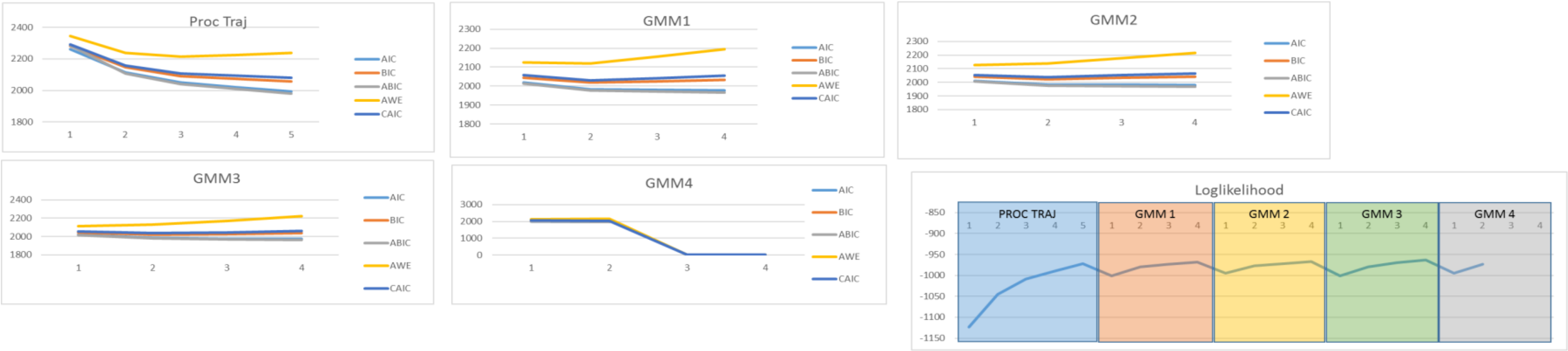

Table 2. Model Information

Candidate Model	Means	Estimate	SE of Estimate	p-value	Residual Variances	Estimate	p-value	Variance	Estimate	p-value	Co-variance	Estimate	p-value
LCGA 2 class	**I**	73.73	1.34	0	**ABC3**	34.92	0						
	**S**	-1.01	1.31	0.439	**ABC4**	37.32	0						
	**Q**	0.35	0.32	0.274	**ABC5**	41.08	0						
					**ABC6**	33.80	0						
					**ABC7**	37.05	0						
	**I**	63.77	1.72	0	**ABC3**	34.92	0						
	**S**	-6.13	1.22	0	**ABC4**	37.32	0						
	**Q**	1.27	0.25	0	**ABC5**	41.08	0						
					**ABC6**	33.80	0						
					**ABC7**	37.05	0						
LCGA 3 class	**I**	70.46	1.36	0	**ABC3**	29.57	0						
	**S**	-2.52	1.33	0.059	**ABC4**	22.81	0						
	**Q**	0.49	0.28	0.081	**ABC5**	23.26	0.001						
					**ABC6**	21.01	0						
					**ABC7**	26.73	0						
	**I**	62.23	1.92	0	**ABC3**	29.57	0						
	**S**	-6.64	1.30	0	**ABC4**	22.81	0						
	**Q**	1.41	0.26	0	**ABC5**	23.26	0.001						
					**ABC6**	21.01	0						
					**ABC7**	26.73	0						
	**I**	75.72	1.15	0	**ABC3**	29.57	0						
	**S**	2.56	2.38	0.281	**ABC4**	22.81	0						
	**Q**	-0.26	0.56	0.644	**ABC5**	23.26	0.001						
					**ABC6**	21.01	0						
					**ABC7**	26.73	0						
GMM1 2 class	**I**	66.06	1.25	0	**ABC3**	19.54	0.001	**I**	33.51	0			
	**S**	-6.58	0.92	0	**ABC4**	19.61	0	**S**	0.00	999			
	**Q**	1.33	0.21	0	**ABC5**	14.41	0	**Q**	0.00	999			
					**ABC6**	3.94	0.12						
					**ABC7**	11.18	0.009						
	**I**	71.69	1.47	0	**ABC3**	19.54	0.001	**I**	33.51	0			
	**S**	1.40	1.23	0.257	**ABC4**	19.61	0	**S**	0.00	999			
	**Q**	0.07	0.38	0.85	**ABC5**	14.41	0	**Q**	0.00	999			
					**ABC6**	3.94	0.12						
					**ABC7**	11.18	0.009						
GMM2 2 class	**I**	71.68	1.46	0	**ABC3**	16.45	0.01	**I**	46.41	0	**I/S**	-3.06	0.287
	**S**	1.22	1.12	0.277	**ABC4**	18.93	0	**S**	0.29	0.67			
	**Q**	0.20	0.31	0.524	**ABC5**	13.62	0	**Q**	0.00	999			
					**ABC6**	5.44	0.06						
					**ABC7**	8.26	0.133						
	**I**	66.03	1.15	0	**ABC3**	16.45	0.01	**I**	46.41	0	**I/S**	-3.06	0.287
	**S**	-6.32	0.80	0	**ABC4**	18.93	0	**S**	0.29	0.67			
	**Q**	1.27	0.17	0	**ABC5**	13.62	0	**Q**	0.00	999			
					**ABC6**	5.44	0.06						
					**ABC7**	8.26	0.133						
GMM3 2 class	**I**	71.70	1.52	0	**ABC3**	19.53	0.001	**I**	33.29	0.006	**I/S**	0.00	999
	**S**	1.40	1.22	0.255	**ABC4**	19.59	0	**S**	0.00	999			
	**Q**	0.07	0.39	0.85	**ABC5**	14.41	0	**Q**	0.00	999			
					**ABC6**	3.96	0.128						
					**ABC7**	11.16	0.009						
	**I**	66.07	1.26	0	**ABC3**	19.53	0.001	**I**	33.63	0	**I/S**	0.00	999
	**S**	-6.57	0.96	0	**ABC4**	19.59	0	**S**	0.00	999			
	**Q**	1.33	0.22	0	**ABC5**	14.41	0	**Q**	0.00	999			
					**ABC6**	3.96	0.128						
					**ABC7**	11.16	0.009						

Table 3. Classification Quality

Candidate Models	Class	Estimated proportion	Entropy	Average Posterior Probability	Odds of Correct Classification (OCC)	Modal Class Assignment Proportion (MCAP)	Homogeneity: I	Homogeneity: S	Homogeneity: Q	Comparison	Separation: I mean	Separation: S mean	Separation: Q mean
LCGA 2 class	1	.39	.84	.96	34.81038	.37				1:2	7.95	4.56	1.76
	2	.61		.95	11.65574	.63							
LCGA 3 class	1	.37	.84	.91	18.09617	.36				1:2	6.37	3.60	1.80
	2	.48		.93	14.17488	.50				1:3	4.61	3.97	1.24
	3	.15		.96	147.4865	.14				2:3	10.24	7.37	2.91
GMM1 2 class	1	.73	.78	.93	5.314118	.76	.72	.10	.10	1:2	4.93	7.95	2.51
	2	.27		.93	37.08332	.24	.63	.10	.10				

Table 4. Correlates of Class Membership

	COHORT	Obs	Variable	N	Mean	Std Dev	Min	Max
Class 1: Low/Decreasing	2	13	NVDQ	13	67.53	11.39	41.44	81.75
			VDQ	13	42.54	17.78	20.13	87.59
			ADOS RRB CSS	12	8.50	1.38	6	10
			ADOS SA CSS	12	7.58	1.78	4	10
			CBCL Internalizing	9	58.78	8.09	49	73
			CBCL Externalizing	9	54.67	10.31	42	73
	3	32	NVDQ	32	59.18	12.59	38.19	86.8
			VDQ	32	39.07	16.38	17.12	78.37
			ADOS RRB CSS	32	8.34	1.38	5	10
			ADOS SA CSS	32	7.16	1.48	4	10
			CBCL Internalizing	27	58.56	6.86	41	72
			CBCL Externalizing	27	54.00	9.68	35	76
	4	47	NVDQ	47	52.04	11.98	27.82	95.71
			VDQ	47	34.35	14.19	15.64	68.89
			ADOS RRB CSS	47	8.15	1.18	5	10
			ADOS SA CSS	47	7.04	1.47	3	10
			CBCL Internalizing	42	60.86	7.91	43	81
			CBCL Externalizing	42	57.00	9.40	39	86
	5	57	NVDQ	56	45.27	13.28	25.41	92.16
			VDQ	56	31.22	12.94	12.49	67.55
			ADOS RRB CSS	56	8.20	1.43	6	10
			ADOS SA CSS	56	7.32	1.43	4	10
			CBCL Internalizing	49	61.27	8.81	41	77
			CBCL Externalizing	49	58.35	8.99	35	80
	6	41	NVDQ	41	44.68	14.87	25.81	88.44
			VDQ	41	30.71	13.48	13.03	74.39
			ADOS RRB CSS	41	8.22	1.11	6	10
			ADOS SA CSS	41	6.93	1.62	3	10
			CBCL Internalizing	36	54.56	7.67	41	71
			CBCL Externalizing	36	54.83	8.32	33	69
	7	45	NVDQ	44	41.60	15.64	20.11	85.64
			VDQ	44	28.14	10.67	11.03	55.32
			ADOS RRB CSS	43	8.51	1.14	6	10
			ADOS SA CSS	43	7.05	1.25	4	10
			CBCL Internalizing	32	55.03	8.97	34	68
			CBCL Externalizing	32	57.13	8.33	33	75
	8	17	NVDQ	17	37.50	13.20	19.39	75.25
			VDQ	17	26.38	10.89	8.71	50.98
			ADOS RRB CSS	17	8.41	1.50	6	10
			ADOS SA CSS	17	7.00	1.22	5	9
			CBCL Internalizing	15	55.60	8.30	41	72
			CBCL Externalizing	15	56.00	7.10	47	71
	9	10	NVDQ	10	41.19	24.37	21.05	105.04
			VDQ	10	28.32	11.88	8.84	52.52
			ADOS RRB CSS	10	8.70	1.06	7	10
			ADOS SA CSS	10	7.40	1.35	5	10
			CBCL Internalizing	7	53.43	10.98	34	65
			CBCL Externalizing	7	59.29	7.06	49	71

Table 4. Correlates of Class Membership

	COHORT	Obs	Variable	N	Mean	Std Dev	Min	Max
Class 2: Moderate/Stable	2	7	NVDQ	5	68.72	9.99	54.26	78.33
			VDQ	5	39.76	9.59	31.31	55.48
			ADOS RRB CSS	6	9.00	0.89	8	10
			ADOS SA CSS	6	7.50	1.64	6	10
			CBCL Internalizing	4	60.00	3.74	55	64
			CBCL Externalizing	4	61.00	3.16	58	65
	3	14	NVDQ	13	74.81	15.37	51.91	108.72
			VDQ	13	65.07	19.17	33.16	93.88
			ADOS RRB CSS	14	7.21	2.33	1	10
			ADOS SA CSS	14	5.79	1.58	2	8
			CBCL Internalizing	12	63.42	3.96	55	69
			CBCL Externalizing	12	60.67	8.47	47	77
	4	21	NVDQ	20	77.66	15.47	56.4	109.18
			VDQ	20	64.71	11.08	42.81	88.48
			ADOS RRB CSS	20	7.45	1.36	6	10
			ADOS SA CSS	20	6.30	1.89	1	9
			CBCL Internalizing	15	60.73	10.48	45	83
			CBCL Externalizing	15	56.53	12.30	42	89
	5	22	NVDQ	21	78.67	17.57	38.3	119.37
			VDQ	21	66.72	16.31	38.3	96.48
			ADOS RRB CSS	21	8.00	1.34	6	10
			ADOS SA CSS	21	7.43	1.57	3	9
			CBCL Internalizing	18	58.61	9.89	43	83
			CBCL Externalizing	18	55.72	10.15	39	89
	6	17	NVDQ	17	88.45	14.32	68.29	118.06
			VDQ	17	76.90	22.59	42.76	118.49
			ADOS RRB CSS	15	7.33	2.47	1	10
			ADOS SA CSS	15	6.60	2.38	2	10
			CBCL Internalizing	14	55.07	10.96	34	67
			CBCL Externalizing	14	51.93	9.06	33	67
	7	10	NVDQ	9	92.85	10.98	77.08	107.71
			VDQ	9	76.44	15.42	53.72	98.24
			ADOS RRB CSS	9	8.00	1.12	7	10
			ADOS SA CSS	9	7.56	2.51	2	10
			CBCL Internalizing	8	51.63	8.37	41	67
			CBCL Externalizing	8	48.75	9.98	33	67
	8	4	NVDQ	4	85.11	18.47	58.25	99.3
			VDQ	4	65.06	10.20	54.21	78.85
			ADOS RRB CSS	4	6.50	3.70	1	9
			ADOS SA CSS	4	6.50	2.65	3	9
			CBCL Internalizing	4	45.25	3.69	41	50
			CBCL Externalizing	4	44.00	13.09	33	63

Table 5. Fit Index Interpretation

Model Fit Index	Interpretation
Classes	Number of classes requested. Is changed by the user and is not an indicator of model quality.
Starts	Number of random starts used to determine the best loglikelihood value. Is changed by the user and is not an indicator of model quality.
Final starting values sets converging	Number of starts that successfully converged. Higher proportion is better (lower suggests instability of the solution).
LL Replication	Number of times the loglikelihood was replicated. Higher is better (lower suggests instability of the solution).
Smallest Class	The size of the smallest extracted class. Small classes may be unstable; must use discretion.
Condition Number	The condition number is the ratio of the smallest to the largest eigenvalue of the estimated information matrix. Very low values imply that the information matrix is singular which implies that the model is not identified.
Loglikelihood	An index of the probability of the data, as a function of the parameters. Algorithm is attempting to maximize this probability. Relative fit statistics are based on this value (and can only be compared within model).
AIC	Mplus calculates as -2LL + 2*r where r is number of free parameters. Smaller values are better.
BIC	Mplus calculates as -2LL + p*ln(N) where p is the number of parameters and N is the sample size. Smaller values are better.
ABIC	Sample size-adjuste BIC; BIC underestimates the number of classes when samples are small. Smaller values are better.
Vuong-Lo-Mendell-Rubin LRT p-value	LRT = Likelihood Ratio Test, which is the difference between LL of model with K classes and K-1 classes. VLMR analytically derives this. Looking for significant p-value to indicate better fit for K classes versus K-1 classes.
Lo-Mendell-Rubin adjusted LRT p-value	LRT = Likelihood Ratio Test, which is the difference between LL of model with K classes and K-1 classes. LMR analytically derives this. Looking for significant p-value to indicate better fit for K classes versus K-1 classes.
Parametric Bootstrap LRT p-value	LRT = Likelihood Ratio Test, which is the difference between LL of model with K classes and K-1 classes. Bootstrap uses an empirically derived distribution of LRT. Looking for significant p-value to indicate better fit for K classes versus K-1 classes.
SIC	SIC = -0.5*BIC, calculated only in service of Bayes' Factor
Bayes' Factor	Bayes' Factor = exp[SIC(Model with K classes) - SIC(Model with K-1 classes)]; approximately equivalent to the ratio of the probability that Model with K is correct to the probability that Model with K-1 is correct. Evidence for Model with K: weak (1-3), moderate (3-10), strong (>10)
AWE	Approximate weight of evidence criterion. AWE=-2LL + 2d[log(n)+1.5], where d is number of parameters and n is sample size.
CAIC	Constant AIC = -2LL +d[log(n)+1], where d is number of parameters and n is sample size.
**Classification Quality Index**	**Interpretation**
Entropy	Index that summarizes the overall precision of classification for the whole sample across the latent classes (>.90 considered excellent)
Average Posterior Probability	Posterior class probability is the model-estimated value for each individual's probability of being in each of the latent classes. APP is the average of these values in individuals with a most-likely class assignment for a given class. Values >0.80 indicate adequate separation and classification precision.
Odds of Correct Classification (OCC)	Ratio of odds of correct classification based on modal class assignment to odds of correct classification based on random assignment. Values >5 indicate adequate separation and precision.
Modal Class Assignment Proportion (MCAP)	Proportion of subjects with modal assignment in class. Compare to model-estimated proportion for consistency.
Homogeneity	Ratio of within-class variance to overall population variance; >0.90 is low homogeneity, <0.60 is high homogeneity
Separation	Degree of overlap of indicator distributions; <0.85 is low separation (high overlap), >2.0 is high separation (low overlap)

NOTE: All information sourced from Masyn, K. (2016). General Growth Mixture Modeling. *Developmental Methods Conference* , Whitefish, MT.

```
TITLE:  Vineland Growth Model
!This is a standard latent growth model. Use to determine shape of trajectory

DATA:   FILE IS all.dat;
        FORMAT IS FREE;

VARIABLE: NAMES ARE abc3 abc4 abc5 abc6 abc7 id;

    IDVARIABLE IS id;
    USEVAR  abc3 abc4 abc5 abc6 abc7;
    MISSING ARE ALL(-999);

ANALYSIS:
    STITERATIONS=10;
    COVERAGE=0.00;

MODEL:
    I S Q| abc3@0 abc4@1 abc5@2 abc6@3 abc7@4 ;
    Q@0;

!Variance of quadratic term is held constant at zero. This is due to small sample size.
!If sample size allows, variance of quadratic term should be estimated
!(as well as covariances where appropriate).
```

```
TITLE:  Vineland Proc Traj
!Mean of intercept, slope, and quadratic term allowed to vary between classes but
!not within

DATA:   FILE IS all.dat;
        FORMAT IS FREE;

VARIABLE:
        NAMES ARE abc3 abc4 abc5 abc6 abc7 id;
        IDVARIABLE IS id;
        USEVAR abc3 abc4 abc5 abc6 abc7;
        MISSING ARE ALL(-999);
        CLASSES = C(1); !change to increase # classes

ANALYSIS:
       type=MIXTURE ;
       MITERATIONS=1000;
       STARTS=500 100;
       STITERATIONS=10;
       COVERAGE=0.00;
       ALGORITHM = INTEGRATION;

MODEL:

        %OVERALL%
        I S Q| abc3@0 abc4@1 abc5@2 abc6@3 abc7@4;

        I@0;
        S@0;
        Q@0;

!Variance of quadratic term is held constant at zero. This is due to small sample size.
!If sample size allows, variance of quadratic term should be estimated
!(as well as covariances where appropriate).

OUTPUT:
        SAMPSTAT STANDARDIZED TECH1 TECH4
        TECH8 TECH11 TECH14 SVALUES;
```

```
TITLE:  Vineland GMM1
!Both means and variance allowed to vary within classes, but intercept
!and slope do not covary

DATA:   FILE IS all.dat;
        FORMAT IS FREE;

VARIABLE:
        NAMES ARE abc3 abc4 abc5 abc6 abc7 id;
        IDVARIABLE IS id;
        USEVAR abc3 abc4 abc5 abc6 abc7;
        MISSING ARE ALL(-999);
        CLASSES = C(1); !change to increase # classes

ANALYSIS:
       type=MIXTURE ;
       MITERATIONS=1000;
       STARTS=500 100;
       STITERATIONS=10;
       COVERAGE=0.00;
       ALGORITHM = INTEGRATION;

MODEL:
        %OVERALL%
        I S Q| abc3@0 abc4@1 abc5@2 abc6@3 abc7@4;

        I*(1);
        S*(2);
        Q@0;

        I WITH S @0;

!Variance of quadratic term is held constant at zero. This is due to small sample size.
!If sample size allows, variance of quadratic term should be estimated
!(as well as covariances where appropriate).

OUTPUT: SAMPSTAT STANDARDIZED TECH1 TECH4
        TECH8 TECH11 TECH14 SVALUES;
```

```
TITLE:  Vineland GMM2
!Means, variance, and covariance of intercept and slope allowed to vary within classes

DATA:   FILE IS all.dat;
        FORMAT IS FREE;

VARIABLE:
        NAMES ARE abc3 abc4 abc5 abc6 abc7 ID;
        IDVARIABLE IS id;
        USEVAR abc3 abc4 abc5 abc6 abc7;
        MISSING ARE ALL(-999);
        CLASSES = C(1); !change to increase # classes

ANALYSIS:
       type=MIXTURE ;
       MITERATIONS=1000;
       STARTS=500 100;
       STITERATIONS=10;
       COVERAGE=0.00;
       ALGORITHM = INTEGRATION;

MODEL:
        %OVERALL%
        I S Q| abc3@0 abc4@1 abc5@2 abc6@3 abc7@4;

        I*(1);
        S*(2);
        Q@0;

        I with S (3);

!Variance of quadratic term is held constant at zero. This is due to small sample size.
!If sample size allows, variance of quadratic term should be estimated
!(as well as covariances where appropriate).

OUTPUT: SAMPSTAT STANDARDIZED TECH1 TECH4
        TECH8 TECH11 TECH14 SVALUES;
```

```
TITLE:  Vineland GMM3
!Variance of intercept and slopes allowed to vary between classes

DATA:   FILE IS all.dat;
        FORMAT IS FREE;

VARIABLE:
        NAMES ARE abc3 abc4 abc5 abc6 abc7 id;
        IDVARIABLE IS id;
        USEVAR abc3 abc4 abc5 abc6 abc7;
        MISSING ARE ALL(-999);
        CLASSES = C(1); !change this to add classes

ANALYSIS:
       type=MIXTURE ;
       MITERATIONS=1000;
       STARTS=500 100;
       STITERATIONS=10;
       COVERAGE=0.00;
       ALGORITHM = INTEGRATION;

MODEL:
        %OVERALL%
        i s q| abc3@0 abc4@1 abc5@2 abc6@3 abc7@4;

        i*;
        s*;
        q@0;

        i with s @0;

        %C#1%
        i*;
        s*;

!Activate these as you add classes
        !%C#2%
        !i*;
        !s*;

        !%C#3%
        !i*;
        !s*;

        !%C#4%
        !i*;
        !s*;

!Variance of quadratic term is held constant at zero. This is due to small sample size.
!If sample size allows, variance of quadratic term should be estimated
!(as well as covariances where appropriate).

OUTPUT:
```

SAMPSTAT STANDARDIZED TECH1 TECH4
TECH8 TECH11 TECH14 SVALUES;

```
TITLE:  Vineland Proc Traj
!All parameters allowed to vary both within and between classes

DATA:   FILE IS all.dat;
        FORMAT IS FREE;

VARIABLE:
        NAMES ARE abc3 abc4 abc5 abc6 abc7 id;
        IDVARIABLE IS id;
        USEVAR abc3 abc4 abc5 abc6 abc7;
        MISSING ARE ALL(-999);
        CLASSES = C(1); !change to increase # classes

ANALYSIS:
       type=MIXTURE ;
       MITERATIONS=1000;
       STARTS=500 100;
       STITERATIONS=10;
       COVERAGE=0.00;
       ALGORITHM = INTEGRATION;

MODEL:
        %OVERALL%
        i s q| abc3@0 abc4@1 abc5@2 abc6@3 abc7@4;

        i*;
        s*;
        q@0;

        i with s *;

        %C#1%
        i*;
        s*;
        i with s*;

!Activate these as you add classes
        !%C#2%
        !i*;
        !s*;
        !i with s*;

        !%C#3%
        !i*;
        !s*;
        !i with s*;

        !%C#4%
        !i*;
        !s*;
        !i with s*;

!Variance of quadratic term is held constant at zero. This is due to small sample size.
```

```
!If sample size allows, variance of quadratic term should be estimated
!(as well as covariances where appropriate).

OUTPUT:
	SAMPSTAT STANDARDIZED TECH1 TECH4
	TECH8 TECH11 TECH14 SVALUES;
```
